# An Image Histogram Equalization Acceleration Method for Field-Programmable Gate Arrays Based on a Two-Dimensional Configurable Pipeline

**DOI:** 10.3390/s24010280

**Published:** 2024-01-03

**Authors:** Yan Wang, Peirui Liu, Dalin Li, Kangping Wang, Rui Zhang

**Affiliations:** 1 Key Laboratory of Symbol Computation and Knowledge Engineering of Ministry of Education, College of Computer Science and Technology, Jilin University, Changchun 130012, China; wy6868@jlu.edu.cn (Y.W.); liupr21@mails.jlu.edu.cn (P.L.);; 2 School of Computer Science, Zhuhai College of Science and Technology, Zhuhai 519041, China

**Keywords:** field-programmable gate arrays (FPGAs), histogram equalization, two-dimensional pipeline, hierarchical state machine

## Abstract

New artificial intelligence scenarios, such as high-precision online industrial detection, unmanned driving, etc., are constantly emerging and have resulted in an increasing demand for real-time image processing with high frame rates and low power consumption. Histogram equalization (HE) is a very effective and commonly used image preprocessing algorithm designed to improve the quality of image processing results. However, most existing HE acceleration methods, whether run on general-purpose CPUs or dedicated embedded systems, require further improvement in their frame rate to meet the needs of more complex scenarios. In this paper, we propose an HE acceleration method for FPGAs based on a two-dimensional configurable pipeline architecture. We first optimize the parallelizability of HE with a fully configurable two-dimensional pipeline architecture according to the principle of adapting the algorithm to the hardware, where one dimension can compute the cumulative histogram in parallel and the other dimension can process multiple inputs simultaneously. This optimization also helps in the construction of a simple architecture that achieves a higher frequency when implementing HE on FPGAs, which consist of configurable input units, calculation units, and output units. Finally, we optimize the pipeline and critical path of the calculation units. In the experiments, we deploy the optimized HE on a VCU118 test board and achieve a maximum frequency of 891 MHz (which is up to 22.6 times more acceleration than CPU implementations), as well as a frame rate of 1899 frames per second for 1080p images.

## 1. Introduction

Histogram equalization (HE) is a very important image preprocessing method that is used to improve the quality of image processing results [1]. It has a wide range of applications in high-precision online industrial detection [2], unmanned driving [3], medical imaging [4], computer vision [5], video processing [6], etc. There are many different versions of HE [7], such as global histogram equalization, average brightness preserving histogram equalization, bucket modified histogram equalization, and local histogram equalization. Global histogram equalization is commonly used, and its main principle is to change the distribution of the whole image’s histogram to a uniform distribution so as to improve the contrast of the image. However, the characteristics of global histogram equalization limit its maximum frame rate in high-speed scenarios, and its throughput should thus be improved.

Multi-core processors are commonly used for accelerating algorithms according to parallel computing theories. However, the accumulation part of HE limits its parallelism, and it is thus mainly implemented in the serial computing of traditional computers, which leads to its low computing performance. Most existing HE acceleration methods, whether run on general-purpose CPUs or dedicated embedded systems, require further improvement in their frame rate to meet the needs of complex scenarios [8,9]. Even when implemented with GPUs, the performance gains are limited [10]. These methods also meet strict power consumption constraints in embedded scenarios [11,12].

In order to achieve better acceleration performance, our work explores optimization methods based on the principle of adapting algorithms to the hardware, taking field-programmable gate arrays (FPGAs) as the accelerating platform. In contrast with other typical parallel computing platforms, such as multi-core CPUs and GPUs, FPGAs have the characteristics of highly customizable parallel computation, high compatibility, and low power consumption [13].

The process of global HE can be divided into the following steps: calculating the histogram of the image, i.e., the frequency of each pixel (which is the number of pixel occurrences divided by the total number of pixels and is denoted by $p_{x}$); calculating the cumulative histogram of the image, i.e., the frequency that is less than or equal to this pixel (denoted by $cdf_{x}$); obtaining converted pixels are according to the cumulative histogram, and the cumulative histogram is multiplied by the largest pixel value (e.g., if the pixel bit width is 8, the largest pixel value is 255); and finally, the original image is converted.

Satisfactory performance cannot be reached by implementing HE on FPGAs directly [14]. In order to improve the performance of HE on FPGAs, it is necessary to optimize the method’s computing architecture and make it more suitable for FPGAs.

Our work first reconstructed the logic of HE to make it suitable for parallel computation, and it was then optimized to be a 2D pipeline architecture so as to improve the FPGAs’ computation speed. We then used three different methods to implement the optimized HE on FPGAs according to different resource utilization strategies. In addition, the performance bottleneck calculations and logic were optimized on the hardware level. We deployed the optimized HE on a VCU 118 test board [15] using PCIe 3.0 communication ports. Compared with a single-core CPU intel i7-8750H, the acceleration ratio of the PCIe3.0*16 interface can be up to 22.6 times higher. Compared with other FPGA-based implementations of a single-input architecture (based on Zynq-XC7Z030) [16], the performance was 43.0 times higher.

In summary, this paper makes the following contributions:

1. We reconstruct HE according to the principle of adapting algorithms to the hardware such that the customized two-dimensional configurable pipeline is able to improve the throughput.
2. We propose a fully configurable computation architecture, and the parameters of HE such as image resolution, pixel bit width, and number of input pixels can be easily customized.
3. We improve the frequencies of the HE implementation through calculation transformation and by pipelining some of the steps of the calculation, such as accumulation and comparison value updates.

## 2. Related Works

Many previous works have been dedicated to accelerating HE. One architecture that has been studied is the “decoder + parallel computing unit” architecture [17]. The input of the decoder is the pixel, and the output is the result corresponding to all pixel values. The number of outputs is $2^{pixel\_bitwidth}$, i.e., when a pixel is considered as an address and input into the decoder, the output bit of the corresponding address and those values less than the address are both 1, and all other bits are 0. Each computing unit receives one output bit from the decoder. If the value of the bit is 0, its own statistical value remains unchanged; if it is 1, its own statistical value increases by 1. After the statistics are finished, each parallel computing unit stores its statistical values into the BRAM sequentially after calculating the converted pixel values. The advantage of this architecture is that the cumulative histogram that corresponds to all pixels can be calculated at the same time instead of calculating the individual histograms first and then the cumulative histogram, which saves time. However, the wiring of this architecture is very complex, and the net delay is incredibly large, so it cannot be run at a particularly high frequency. This method calculates division using the higher-order bits, and it has limited application scenarios. The results obtained from the calculation need to be sequentially stored in BRAM, which also causes greater computational latency and degrades performance.

A few other articles have applied a similar architecture [18,19]. There are also articles that improve upon this architecture or add other features. For example, the generated histogram can be displayed directly through the display module [16]. An alternative to sequential BRAM writing is to use a multiplexer, which does not require BRAM writing time, while also allowing for the random selection of the calculation results [20]. The calculation of division can also be made more flexible. One way to achieve this is to use an integer divider, but this method cannot compute rounding [21]. The other method is to multiply by a factor and then take the higher-order bits, but this method needs sufficient precision [20].

Another typical architecture is to use BRAM for histogram calculation [22]. The input pixel is used as the BRAM address, and the data stored in the BRAM correspond to the accumulated number of corresponding pixels. But because one of BRAM’s ports can only be read or written in a clock cycle, different studies have used different methods. One such method is to read BRAM only when there is a change in the data. Another method is to use two-port BRAM and read on one port and write the calculated value on the other port, so that the read and write operations can be completed in one clock cycle. After the histogram statistics are completed, the accumulated circuit is used to calculate the accumulated histogram, and the converted data are calculated by the corresponding calculation and stored back in the BRAM. This architecture has the advantage of saving logical resources, but it is limited both in terms of speed and multi-input scaling.

There are many other implementations based on this architecture. Among them, the addition used to generate the histogram can be calculated by DSP, thus reducing the utilization of resources [23]. DSP can also be used in processing histograms. Not only can DSP be used to generate cumulative histograms, but it can also be programmed to achieve additional functions [24]. Based on this architecture, FPGAs and computer software can be combined to achieve heterogeneous computing with greater flexibility that can achieve more functions [25].

For the calculation of HE with multiple inputs and outputs, both of the above architectures have their corresponding variants. Multi-input and -output architectures, such as the multi-decoder architecture, are the first kind [26]. In this artchitecture, multiple pixels are input to multiple decoders, and the output of the same bit from different decoders is input to the same computing unit. Each computing unit adds the accumulated value of its own through the number of 1s obtained from multiple inputs, so as to calculate the accumulated histogram. The second type of multi-input and -output architecture, such as the multi-BRAM port architecture [27,28], can read or write in parallel, but not simultaneously. In the case of parallel writing, statistics are performed through multiple BRAM ports, and each port inputs a part of the image; then, the generated histograms are added together to generate a complete histogram. In the case of parallel reading, one pixel is input each time to multiple BRAM ports, and then multiple identical histograms will be generated and read through multiple BRAM ports, so as to realize parallel reading. The performance of these architectures is not high; the multi-decoder’s wiring is too complex, resulting in a large wiring delay; the multi-BRAM ports do not achieve simultaneous input and output results; and with the increase in the use of BRAM, the performance of the circuit will be reduced.

Concerning the calculation method of the cumulative histogram, some studies replace division by taking the higher-order bit [17] because when the lower-order bits are all 0, directly abandoning the low position is equivalent to a right shift operation, which can replace division. However, this method can only calculate the division when the divisor is the index of 2. Other methods do not optimize the division and use hardware DSPs or configurable logical resources to compute the division.

In addition to designing the computational architecture, the MATLAB code can be automatically converted to RTL code and run on FPGAs [29]. There are also some implementations developed using HLS [30,31] and other tools [32]. These methods may speed up development, but they are not specifically optimized for performance.

## 3. Parallelization of Histogram Equalization and Its Hardware Computing Architecture

### 3.1. Histogram Equalization Algorithm

Each image can be represented as an $m_{r}*m_{c}$ matrix of integer pixel intensities ranging from 0 to L−1. *L* is the number of pixel intensities; if the bit width of the pixel intensities is *w*, then *L* is $2^{w}$. For example, if *w* is 8, *L* is 256.

Let *p* denote the normalized histogram of the image with a bin for each possible intensity, as follows:

(1) $$p_{x}=\frac{n_{x}}{n}=\frac{\sum_{i=1}^{n}\left(x_{i}==x\right)}{n},$$

where $n_{x}$ is the number of pixels with an intensity of *x*; *n* is the total number of pixels and $n=m_{r}*m_{c}$; and $x_{i}$ is the intensity value of the *i*’th pixel of the image, where $\left(x_{i}==x\right)$ is a Boolean expression that equals 1 if $x_{i}$ and *x* are equal and 0 if they are not.

The output pixel intensity of HE for each input pixel intensity *x* is $y_{x}$ and is defined as

(2) $$y_{x}=round\left(\left(L-1\right)\sum_{i=0}^{x}p_{i}\right),$$

where round() rounds to the nearest integer.

### 3.2. Parallelizing Histogram Equalization

We parallelize HE using two approaches: (1) computing all $cdf_{x}$ in parallel and (2) processing multiple input pixels simultaneously.

For processing *m* inputs simultaneously and calculating $\sum_{i=0}^{x}p_{i}$ directly, according to Formula (1),

(3) $$n_{x}=\sum_{i=1}^{n/m}\sum_{j=1}^{m}\left(x_{ij}==x\right),$$

where $x_{ij}$ is the *j*’th input in the *i*’th multiple input.

In addition to computing *m* inputs simultaneously, we can also compute the $cdf_{x}$ directly in parallel. According to Formulas (1)–(3),

(4) $$cdf_{x}=\frac{1}{n}\sum_{k=0}^{x}\sum_{i=1}^{n/m}\sum_{j=1}^{m}\left(x_{ij}==k\right).$$

We can adjust the order of accumulation as follows:

(5) $$\frac{1}{n}\sum_{k=0}^{x}\sum_{i=1}^{n/m}\sum_{j=1}^{m}\left(x_{ij}==k\right)\Rightarrow \frac{1}{n}\sum_{i=1}^{n/m}\sum_{j=1}^{m}\sum_{k=0}^{x}\left(x_{ij}==k\right).$$

The innermost accumulation can be obtained directly:

(6) $$\sum_{k=0}^{x}\left(x_{ij}==k\right)\Rightarrow \left(x_{ij}<=x\right).$$

Finally, the formula to calculate $cdf_{x}$ in parallel is

(7) $$cdf_{x}=\frac{1}{n}\sum_{i=1}^{n/m}\sum_{j=1}^{m}\left(x_{ij}<=x\right),$$

and according to Formulas (2) and (7), f(x) is

(8) $$f\left(x\right)=round(\frac{L-1}{n}\sum_{i=1}^{n/m}\sum_{j=1}^{m}\left(x_{ij}<=x\right)),$$

$\frac{L-1}{n}$ is a constant when *L* and *n* are determined.

So far, HE has been parallelized to fit *m* inputs simultaneously and can calculate $cdf_{x}$ directly in parallel. The parallelized HE involves the computation of three dimensions. Dimension *i* is the time dimension, which is executed sequentially. Dimension *j* is used for multiple inputs, executed in parallel. The *x* dimension is the transformation result of each pixel’s intensity, which is executed in parallel.

### 3.3. Principles of Hardware Computing Architecture

The typical architectures mentioned in the Related Works Section have their own disadvantages and limited performance. The architecture based on BRAM, which is treated as a lookup table, is not capable of parallel input and parallel output at the same time. In addition, the fan-out architecture has a very high net delay and clock skew.

The overall architecture should keep the “total fan out” low. The connection complexity of the architecture with a “high total fan-out” is big, which means that every cell extended in one dimension needs to connect all the cells in the other dimension. As a result, the architecture is not easy to scale, and has high net delay and clock skew.

### 3.4. Architecture for Parallelized Histogram Equalization

**Figure 1:**
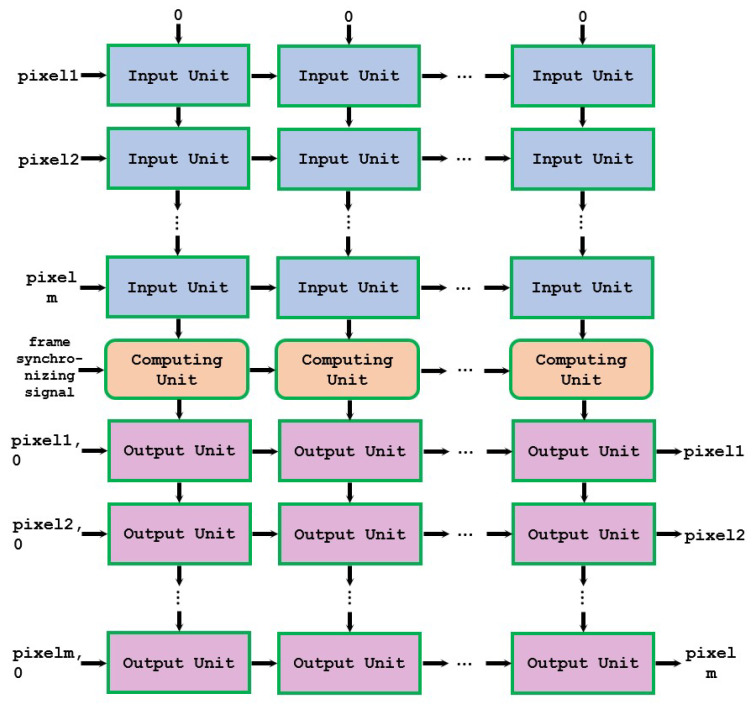
Two-dimensional configurable pipeline architecture.

We proposed a novel approach to realize parallel computing in both dimensions, thus forming the overall architecture of a two-dimensional pipeline, as shown in Figure 1.

The overall architecture consists of input units, computing units, and output units. The input unit inputs the pixels of the image to be computed. When the input pixel (from left) is less than or equal to the pixel for which the input unit is responsible, it will increase the value of the unit above and output the result to the unit below. The input pixels will be directly output to the right.

The behavior of the input units is shown in Algorithm 1, where “pixelIn” is the input from the left, “valueIn” is the input from above, “pixelOut” is the output to the right, and “valueOut” is the output below.
**Algorithm 1** The Behavior of The Input Units**Input:** pixelIn,valueIn **Output:** pixelOut,valueOut    pixelOut ← pixelIn    **if** (pixelIn <= compareNum) **then**        valueOut ← valueIn + 1    **else**        valueOut ← valueIn    **end if**

The computing unit performs multiplication and rounding calculations based on the values input from above, and each computing unit is responsible for the calculation of one pixel’s intensity. The computing unit passes the result to the output unit below. The input from the left is the frame-synchronizing signal, which will control the output of the computing unit and is also directly output to the right.

The behavior of the computing units is shown in Algorithm 2, where “frameSynIn” is the input from the left, “valueIn” is the input from above, “frameSynOut” is the output to the right, and “valueOut” is the output below.
**Algorithm 2** The Behavior of the Computing Units**Input:** valueIn,frameSynIn **Output:** valueOut,frameSynOut    frameSynOut ← frameSynIn    **if** (!frameSynIn) **then**         valueOut ← valueOut         accumulator ← accumulator + valueIn    **else**         valueOut ← $round(\frac{L-1}{n}\;*$ accumulator)         accumulator ← 0    **end if**

After the pixels of a frame are made into input units and the values are calculated by the computing units, the calculation of f(x) in the histogram equalization is completed. At this point, these pixels need to be made into output units to complete their conversion. The output unit of each column is responsible for the transformation of the same pixel, that is, *x* in Equation (8). f(x) is the input from the computing unit above and is output directly below. The pixel to be converted is input from the left, and if the input value is the unit the pixel is responsible for and has not been converted before, the value will be converted, and the result will be output to the right. A flag bit is also required to indicate whether the pixel has been converted. If the pixel has been converted, the flag bit will be set to one.

The behavior of the output units is shown in Algorithm 3, where “pixelIn” is the input from the left, “valueIn” is the input from above, “pixelOut” is the output to the right, and “valueOut” is the output to below. Moreover, “comparedIn” is the flag bit input from the left, and “comparedOut” is the flag bit output to the right.

Each unit is only connected to up to four other units, reducing the connection complexity. The input–output of the units at the edge are special, with and input of 0 for the topmost input unit and an input of 0 for the flag bit of the leftmost output unit. In this way, we can add as many rows or columns as we need without affecting the connections between original units. This means that we can easily scale up the computing architecture without affecting the overall running frequency.
**Algorithm 3** The Behavior of The Output Units**Input:** pixelIn,comparedIn,valueIn **Output:** pixelOut,comparedOut,valueOut    valueOut ← valueIn    **if** (pixelIn == compareNum)&&(!comparedIn) **then**         pixelOut ← valueIn         comparedOut ← 1    **else**         pixelOut ← pixelIn         comparedOut ← comparedIn    **end if**

**Figure 2:**
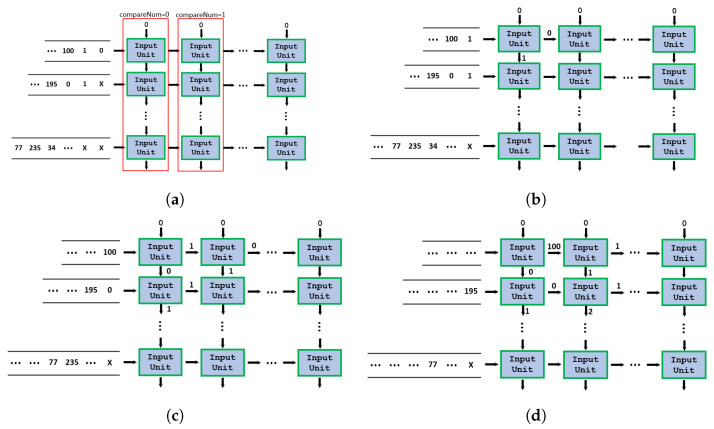
The data flow of input units. (**a**) t = 0. (**b**) t = 1. (**c**) t = 2. (**d**) t = 3.

The example in Figure 2 shows the data flow of input units.

The time complexity of the optimized HE is

(9) O(n)=n/m,

where *m* is the number of inputs and *n* is the number of pixels in the image. The number of units *N* is

(10) N=(2m+1)∗L.

## 4. Implementation of Histogram Equalization on FPGAs

We deploy the optimized HE on FPGAs. In order to study the acceleration effects and resource utilization of HE on FPGAs in depth, we implement HE through three different strategies: (1) with configurable logical (CL) resources only; (2) with CL and DSPs; and (3) with CL, DSPs, and BRAMs. Each strategy achieves a different performance and resource utilization, and can be applied in different scenarios. If we need high speed, we can use the CL only strategy; if the resources of the FPGAs are insufficient, the strategy utilizing CL and DSPs can be used; and if the speed of the interface is limited, the strategy utilizing CL, DSPs, and BRAMs can be used.

### 4.1. Utilizing Only Configurable Logical Resources

This method uses configurable logical resources, except for the FIFO used as the axis interface.

#### 4.1.1. Overall Architecture Based on FPGAs

**Figure 3:**
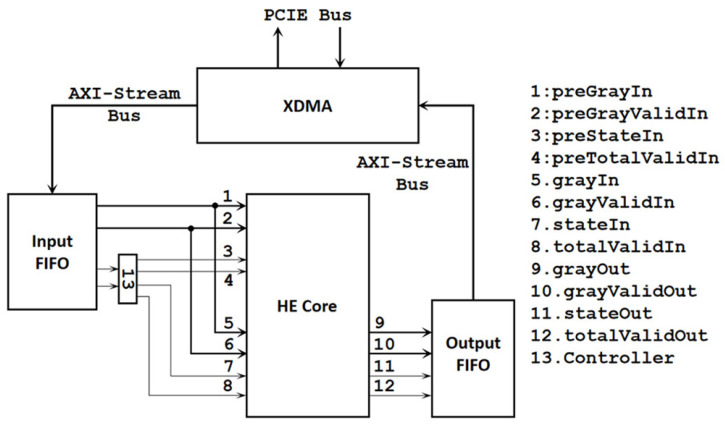
Overall architecture utilizing only CL.

The overall architecture based on FPGAs is shown in Figure 3. The calculation process is to input two identical frames into the InputFIFO successively. The first frame is used for calculation, and the second frame is used for conversion. The Controller controls the input of the first frame to the first set of ports (1, 2, 3, 4) and the input of the second frame to the second set of ports (5, 6, 7, 8).

#### 4.1.2. Overall Architecture of HE Core

**Figure 4:**
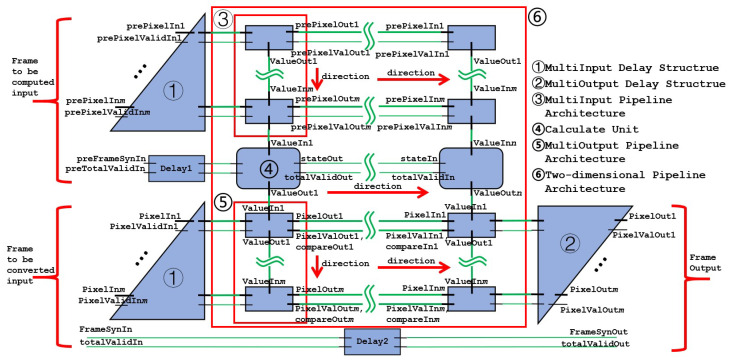
Overall architecture of HE core.

The overall architecture adopted is shown in Figure 4. In the case of increasing input and output, using a pipelined architecture to transfer multiple inputs and outputs can reduce wiring complexity and logic delay and improve performance.

A vertical row of input units constitutes a multi-input pipeline architecture. The final result of the multi-input pipeline architecture represents the number of input pixels that this computing unit is responsible for. The calculate unit takes in the input values, performs calculations, and outputs the final transformed result of the pixel it is responsible for. The calculate unit also receives the state signal, which is used to perform frame synchronization. A vertical row of output units constitutes a multi-output pipeline architecture. The “direction” in the figure indicates the direction of the data flow. The multi-input pipeline architectures, computational units, and multi-output pipeline architectures make up the two-dimensional pipeline architecture.

#### 4.1.3. State Machine Design

**Figure 5:**
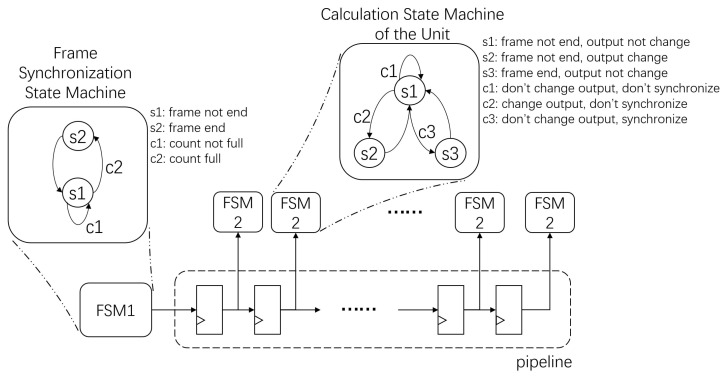
State machine link.

Since this architecture is composed of many computing units, sharing a global state increases the total fan-out degree of the state machine. Different from the global state machine, we adopt a pipelined distributed hierarchical state machine link, as shown in Figure 5.

The first layer’s state machine is used to perform frame synchronization. Here, the state machine is at the head of the pipeline, and the output state is transmitted downstream along the pipeline. The second layer’s state machine is used to indicate the state of the computed histogram, and each cell has its own second-layer state machine.

In the frame synchronization state machine, the state of s1 is in the frame, at which point the counter counts the number of pixels received. The state of s2 is the end of the frame, at which point the counter is cleared. c1 is the transition condition from s1 to s1 when the counter has not reached the end of a frame. c2 is the transition condition from s1 to s2 when the counter reaches the end of a frame.

In the calculation state machine, s1 is the state that counts and keeps the output value, s2 counts and changes the output value, and s3 outputs the output value. c1 represents the condition when the output value should not be changed, c2 is when the output value should be changed but the frame does not end, and c3 is when the frame ends.

Benefiting from the Xilinx’s distributed clock, the latency between blocks is very low, so we use a distributed state machine architecture, where each state machine is measured at the nearest computation unit and takes the clock signal from the block clock, so that the different state machines can maintain a high degree of synchronization. When the number of processing units increases, the maximum frequency caused by the extension of the state machine line will not decrease as the single state machine does.

#### 4.1.4. Details of Some Units

**Figure 6:**
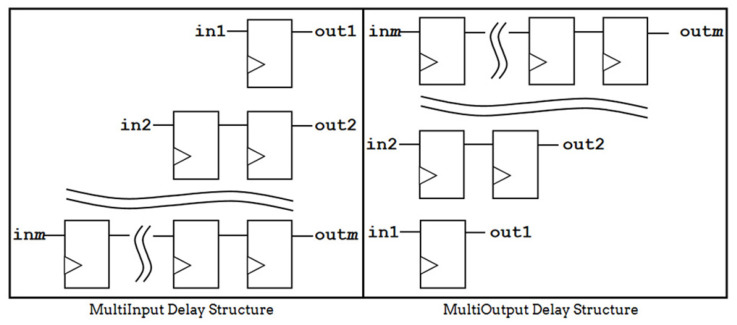
Multi-input/output delay structure.

Since the input and output pipeline architecture needs to output the result of the input in the same clock cycle, there will be a delay structure at the input and output to ensure that the final output of the pipeline architecture is the result from the same clock cycle input. The multi-input and multi-output delay structure is shown in Figure 6.

#### 4.1.5. Calculation Method Optimization

After we calculate $\sum_{i=1}^{n/m}\sum_{j=1}^{m}\left(x_{ij}<=x\right)$ in Equation (7), we need to calculate f(x) in Equation (8). This requires the use of multiplication and division, but we can optimize these functions to addition and subtraction.

For multiplication by L−1, *L* is the exponent of 2, and multiplication by the exponent of 2 can be solved by simply shifting bits to the left, as follows:

(11) x∗(L−1)⇒(x<<w)−x,

where *w* is the pixel bit width. In this way, we optimize multiplication to subtraction.

In the case of dividing by *n* in Equation (8), it can be compared with *n* when accumulating using an accumulation register. If the result is greater than *n*, the result register is incremented by 1, and the accumulated result is reduced by *n* and stored back in the accumulation register. Thus, Algorithm 4 can be optimized to Algorithm 5.
**Algorithm 4** Dividing by *n***Input:** valueIn **Output:** result  1: accumulator ← 0  2: **loop**  3:     accumulator ← accumulator + valueIn  4: **end loop**  5: result ← accumulator/*n*

**Algorithm 5** Optimized Dividing by *n***Input:** valueIn **Output:** result  1: accumulator ← 0  2: result ← 0  3: **loop**  4:     accumulator ← accumulator + valueIn  5:     **if** (accumulator > n) **then**  6:         result ← result + 1  7:         accumulator ← accumulator − *n*  8:     **end if**  9: **end loop**


In this way, we optimize division to addition and subtraction.

By integrating the above two methods, we can obtain an optimized calculation method. The optimized HE is shown in Algorithm 6.
**Algorithm 6** The Behavior of the Computing Unit**Input:** valueIn,frameSyn **Output:** valueOut  1: **if** (!frameSyn) **then**  2:     valueOut ← valueOut  3:     **if** (accumulator + (valueIn << w) − valueIn ≥ n) **then**  4:         accumulator ← accumulator + (valueIn << w) − valueIn − n  5:         result ← result + 1  6:     **else**  7:         accumulator ← accumulator + (valueIn << w) − valueIn  8:         result ← result  9:     **end if** 10: **else** 11:     valueOut ← result 12:     accumulator ← n/2 13:     result ← 0 14: **end if**


The computing unit receives the number from the input module and multiplies the received number by L−1. The value obtained after multiplication is added to the previous value in the accumulation register and is compared with *n*. Then, the accumulator and the result are updated according to the method above.

#### 4.1.6. Critical Path Optimization

The optimized calculation method does not run at very high frequencies, so the critical path needs to be optimized. Here are some optimizations for the critical path:

1. Increase the number of stages in the pipeline: As Algorithm 7 shows, updating *a* in one clock cycle does not result in strong performance for the following computation.
  **Algorithm 7** Updating *a* in one clock cycle  1:**if** (a + b > c) **then**  2:     a ← a + b − c  3:**else**  4:    a ← a + b  5:**end if**
  We can split this algorithm into three stages. The first stage computes a+b, the second stage compares the result to *c* and calculates the difference from *c*, and the third stage selects a result and returns it to *a*.
2. Replace comparison with signed subtraction: Since the comparison operation circuit will degrade the performance degradation of Algorithm 7, the subtraction operation needed at the same time can be changed into a signed subtraction operation, and the sign of the subtraction operation can be used instead of the output of the comparison operation, so that the comparison and subtraction can be performed at the same time, thus improving performance.
3. Replace signed subtraction with addition: The binary subtraction x∗L−x is calculated as
  (12) x∗L−x=x∗L+∼x+1,
  and when we put 0s in the lower-order bits of x to obtain x∗L and then replace the lowest bit of x∗L with 1, we obtain x∗L+1 directly:
  (13) $$x*L-x=\{x,{\left(\$size\left(L\right)-1\right)}^{\prime }b0,1^{\prime }b1\}+∼x.$$
  The above equations refer to Verilog syntax.
4. Use clock enable: The clock enable port can be utilized to simplify the conditional branch circuit and thus improve performance. Clock enable is conducted by establishing the condition that the output value does not change in the outermost layer. This was also on the critical path before optimization.
5. Condition separation: Some state machines have too many states, in which case the condition can be separated into two registers to reduce the signal delay.
6. Loop structure optimization: The feedback circuit with only finite instances of feedback can be optimized into multiple non-feedback circuits with the same structure in series to improve the performance.

The pseudocode of the computing unit with the critical path optimization applied is shown in Algorithm 8, where “q1” is the first digit of the shift register, “q2” is the second digit of the shift register, and “qk” is the k’th digit of the shift register. The input to the shift register is “~frameSyn”. Algorithm 8 refers to Verilog syntax.
**Algorithm 8** The Behavior of the Computing Unit**Input:** valueIn,frameSyn **Output:** valueOut  1: valueIn255 ← {valueIn,8’h01} + {8’hFF,~value}  2: **if** (q3&q4&q5) **then**  3:     stage1 ← valueIn255 + remainder  4: **else**  5:     stage1 ← valueIn255  6: **end if**  7: stage2 ← stage1  8: stage2diff ← stage1 − pixelNum  9: **if** (stage2diff[sign_bit]) **then** 10:     remainder ← stage2 11: **else** 12:     remainder ← stage2diff 13: **end if** 14: **if** (stage2diff[sign_bit]&q4) **then** 15:     quotient ← quotient 16: **else** 17:     **if** (stage2diff[sign_bit]) **then** 18:         quotient ← 0 19:     **else** 20:         quotient ← quotient + 1 21:     **end if** 22: **end if** 23: **if** (q4) **then** 24:     quotientReg ← quotient 25: **else** 26:     quotientReg ← quotientReg 27: **end if** 28: **if** (q1)|(q2&q3&q4) **then** 29:     **if** (q1) **then** 30:         accumulator ← accumulator + remainder 31:     **else** 32:         accumulator ← pixelNum/2 33:     **end if** 34: **else** 35:     accumulator ← accumulator 36: **end if** 37: accudiff1 ← accumulator − pixelNum 38: accudiff2 ← accudiff1 − pixelNum 39: accudiff3 ← accudiff2 − pixelNum 40: **if** (q8) **then** 41:     valueOut ← ~accudiff1 + ~accudiff2 + ~accudiff3 42: **else** 43:     valueOut ← valueOut 44: **end if**


#### 4.1.7. The Architecture of the Computing Unit

**Figure 7:**
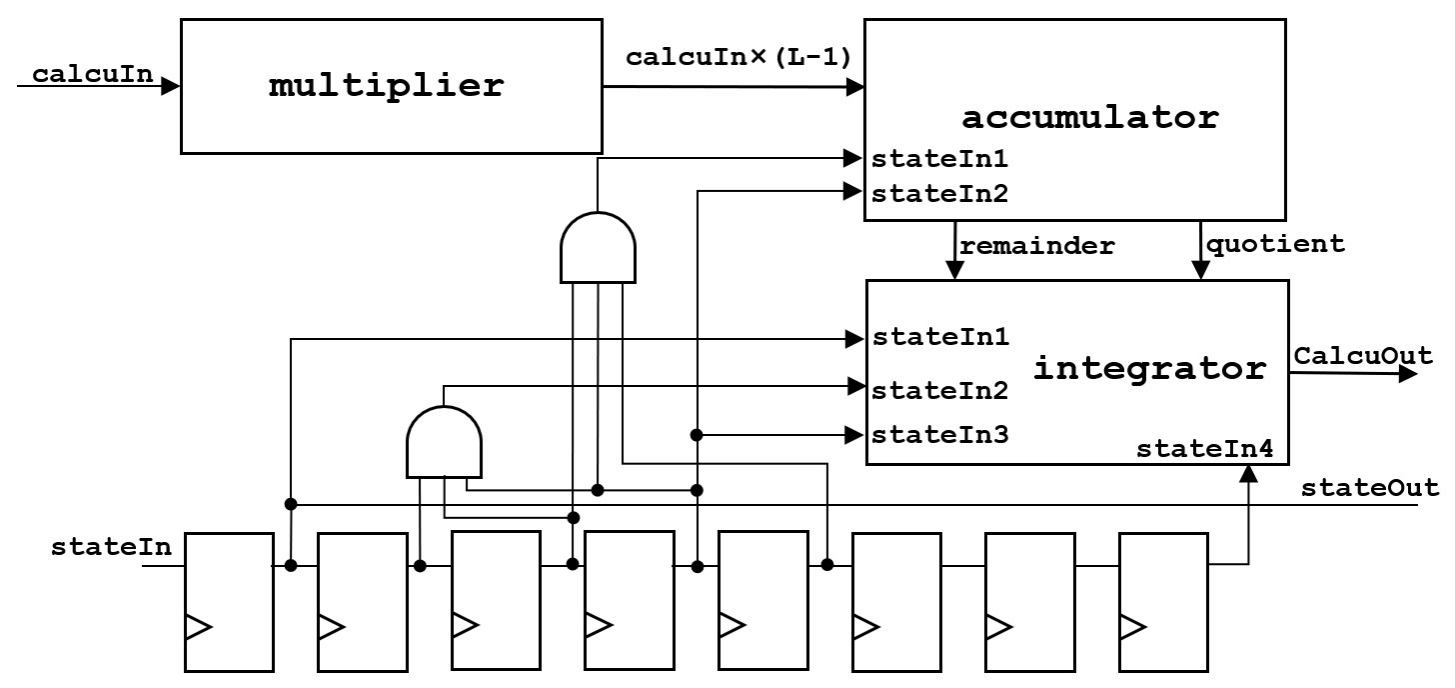
The architecture of the computing unit.

The architecture of the computing unit is shown in Figure 7.

The statistical value of pixels calcuIn will be input to multiplier for multiplication and then input to accumulator for accumulation. The accumulative value will be compared with the total number of pixels. If the value is greater than the total number of pixels, the total number of pixels will be subtracted. Due to the fact that the accumulator accumulative value is updated in three cycles, it will output one quotient and three remainders. Thus, in the end, it will input into integrator, add the remainder of the three groups together, subtract and compare with the total number of pixels three times, and add the result to the quotient to obtain the final result. Moreover, stateIn is the frame-synchronization signal.

**Figure 8:**
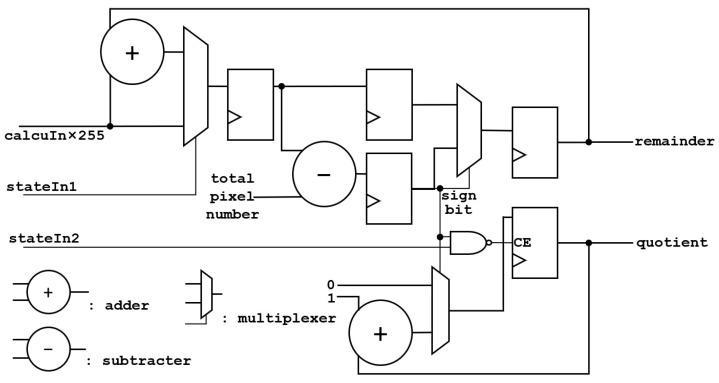
The architecture of the accumulator.

The architecture of the accumulator is shown in Figure 8.

The value after multiplication is input into the accumulator, and the quotient and remainder are calculated. The calculation takes three clock cycles, and each cycle has an input, so the accumulator outputs three remainders. Since all quotients are added to one register, only one quotient is output. stateIn1 is an AND logic with stateIn’s clock delay of three, four, and five cycles, which is an indication signal for the accumulator to reaccumulate at the beginning of a frame. stateIn2 is an indicator signal that the accumulator can clear the quotient.

**Figure 9:**
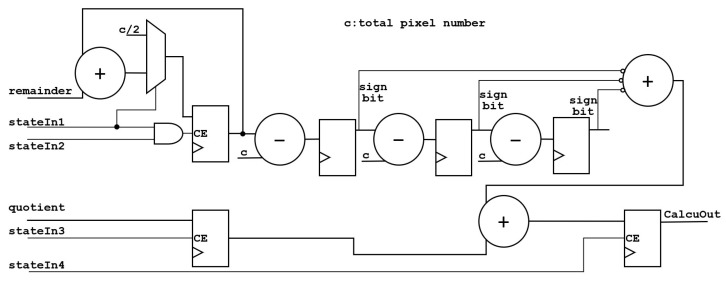
The architecture of the integrator.

The architecture of the integrator is shown in Figure 9.

The integrator adds the three remainders of the input together, compares them with the total number of pixels three times, and adds the three results into the quotient to obtain the final result. stateIn1 is an indication signal for the initialization of the integrator, stateIn2 is the indication signal of the three accumulations, stateIn3 is the indication signal to add the result of the three subtractions and the quotient, and stateIn4 is an indication signal that the integrator outputs the result.

### 4.2. Utilizing CL and DSP

The overall architecture of this method is the same as the previous method, but the configurable logic resources responsible for the computation in the computing unit are replaced with DSP. The accumulation and division functions of the unit are completed by DSP. To calculate the rounding value of a number multiplied by a fraction, we can use DSP’s integer multiplication and accumulation:

(14) $$round\left(x*\frac{a}{b}\right)=x*\left\lfloor \frac{a*2^{\$size(b)+accuracy-1-\$size(a)}}{b}\right\rfloor ,$$

where *b* is 2 to the *n* and dividing by *b* takes the higher-order bits. We take 9 higher-order bits and round them to the 8-bit result. Thus, the approximate division operation is realized. When the accuracy of the calculation is sufficient, there is no error.

In order to satisfy the accuracy, we use two DSPs to calculate the multiplication with a bit width of 34 bits. One DSP is used to compute the multiplication and accumulation operations of the lower-order bits, and the other DSP is used to compute the multiplication and accumulation operations of the higher-order bits, and then add the output value from the low-order bits.

### 4.3. Utilizing CL, DSP, and BRAM

**Figure 10:**
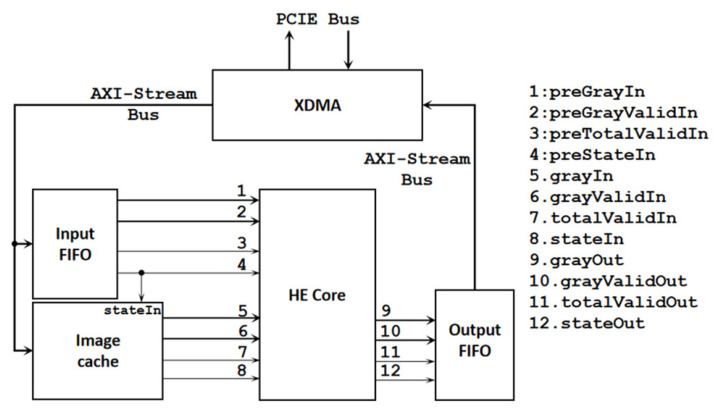
Overall architecture utilizing CL, DSP, and BRAM.

Based on utilizing DSP resources, this method adds an image cache implemented by BRAM. This architecture does not require the same image to be input twice. The overall architecture is shown in Figure 10. The frame to be converted will be stored in the image cache and be output according to the current state.

## 5. Experiments

### 5.1. Experimental Environment

The VCU 118 evaluation board platform was used in this experiment. The PCIe interface of the evaluation board is PCIe3.0*16. The FPGA on the VCU118 contains 2.6 million logical units, 6840 DSPs, and 2160 Block RAM, with a total capacity of 75.9 MB. The chip has a 16 nm fabrication process, the maximum frequency of the clock buffer and DSP is 775 MHz, and the maximum frequency of the Block RAM is 737 MHz. The XDMA AXI side operates at 250 MHz.

### 5.2. Experimental Results

**Figure 11:**
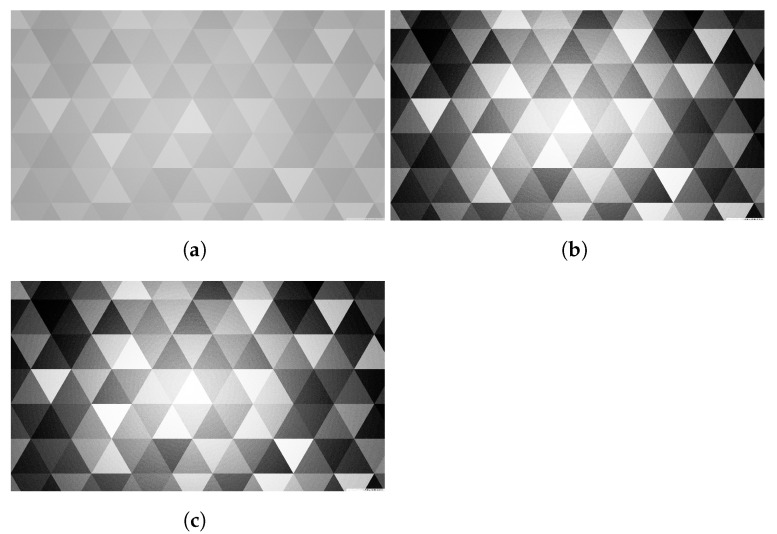
(**a**) Figure before histogram equalization. (**b**) Figure after histogram equalization with FPGAs. (**c**) OpenCV histogram equalization output image.

Figure 11 shows the picture before conversion and the picture after conversion with FPGAs. The converted image is consistent with the openCV’s histogram equalization output image, which proves that the running result is correct.

### 5.3. Resource Utilization of the Implementations with Different Parameters

This experiment tests the resource utilization of the three architectures with different resolutions and different numbers of PCIe lanes.

**Figure 12:**
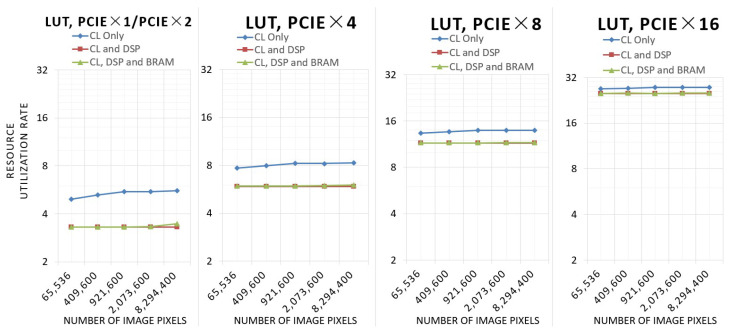
Resource utilization of LUT.

Figure 12 shows the resource utilization of LUT.

As the number of pixels rises, the LUT utilization of the architecture using only configurable logic (CL) increases, while the LUT utilization of the other two architectures utilizing DSP remains basically the same. Due to the fact that a larger number of image pixels requires a wider bit width calculation when utilizing only CL, the number of DSPs and the computing bit width utilizing CL and DSP and utilizing CL, DSP, and BRAM remain the same. In the architecture utilizing CL, DSP, and BRAM, the utilization of LUTs will be slightly higher at 4 k resolution because a portion of the LUTs will be used to route FIFOs with high BRAM utilization.

**Figure 13:**
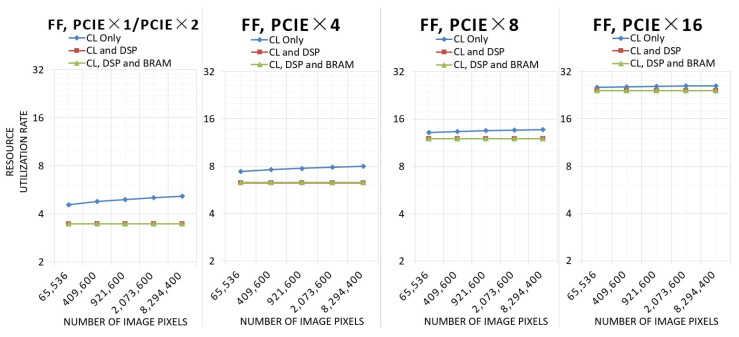
Resource utilization of flipflop.

Figure 13 shows the resource utilization of flipflop.

The situation of flipflop utilization is similar to that of LUT utilization. The architecture utilizing only CL uses more flipflops as the computational bit width increases, while the other two architectures are unchanged. However, the architecture utilizing CL, DSP, and BRAM does not need to use more flipflops at 4k resolution, so the utilization of flipflops will not increase.

**Figure 14:**
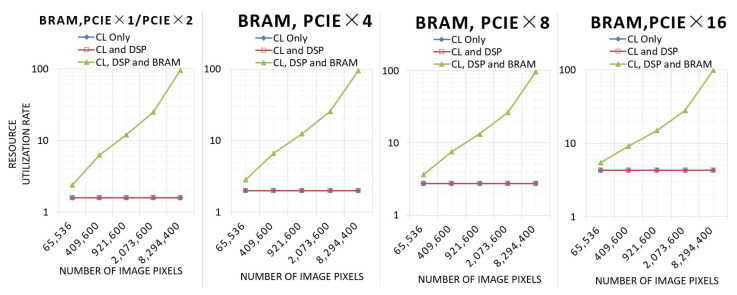
Resource utilization of BRAM.

Figure 14 shows the resource utilization of BRAM.

The architecture using only CL and the architecture using CL and DSP hardly utilize any BRAM resources, while the BRAM utilization of architectures using CL, DSP, and BRAM will increase significantly with the increase in the number of pixels.

As the number of PCIe channels increases, the LUT, FlipFlop, and BRAM utilization of the three architectures increases. However, the utilization of DSP for the architecture utilizing CL and DSP and the architecture utilizing CL, DSP, and BRAM is 7.49%, independent of the number of inputs and the total number of pixels.

### 5.4. Performance Comparison with CPU and Other FPGA-Based Implementations under Different Configurations

#### 5.4.1. Comparison with Single-Core CPU

The CPU model is an i7-8750H, and the HE is realized using the OpenCV framework. The OpenCV framework uses only one core of the CPU. This performance test method measures the difference between the running time of 11 images and 1 image, divides by 10 to obtain the running result of each image, and takes the average result of 10 tests.

**Figure 15:**
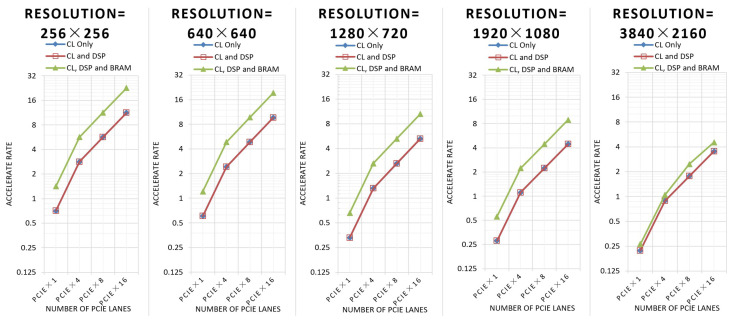
Acceleration rate for a single-core CPU.

Figure 15 shows the acceleration rate of this implementation for a single-core CPU.

When the resolution of the image is 3840 × 2160, in the architecture using CL, DSP, and BRAM, the BRAM utilization rate is close to 100%, so the running frequency will be decreased, and the performance will not be doubled compared with the other two architectures.

#### 5.4.2. Comparison with Other FPGA-Based Implementations

A comparison with other FPGA-based implementations is shown in Table 1. The acceleration rate is the performance of proposed implementation divided by the performance of other FPGA-based implementations. Acceleration ratios take into account the impact of different models on performance.

Compared to the fastest single-input FPGA-based implementation [16], the performance of the architecture utilizing CL and the architecture using CL and DSP is about 21.4 times better, and the performance of the architecture utilizing CL, DSP, and BRAM is about 43.0 times better (65,536 pixels under PCIe×16).

Other architectures with multiple inputs do not mention a specific frequency, so we consider them architecturally. The architecture in [26] is difficult to run at very high frequency due to the need to connect modules at a long distance, the high fan-out degree, and the complex wiring. The architecture in [27,28] does not really support multiple inputs and outputs at the same time.

The previous comparisons are based on works accelerating the same algorithm, but on different hardware platforms, as they are finished at different times. There is also work using the ultrascale+ architecture of FPGAs [33], which implements the Contrast-Limited Adaptive Histogram Equalization (CLAHE) algorithm. In this work, the authors redesigned a vector stream format (4 ppc) to implement the CLAHE algorithm, which enables the processing of a 4K video stream at 60 fps. Our work can reach 964 fps for 4K images. This difference is mainly caused by the difference in complexity between the two algorithms. We both designed optimized FPGAs architectures for accelerating CLAHE and HE. Therefore, users can select which one to use according to their requirements. In addition, the innovative methods in [33] can also be references for our future research.

## 6. Discussion and Conclusions

In order to improve the calculation performance of HE, we optimized HE according to the characteristics of FPGAs and proposed a two-dimensional pipeline architecture which parallelizes HE in two dimensions. At the same time, the complexity of the connection is not high, so the FPGAs can run at a high frequency. The optimized HE is implemented on FPGAs, and the number of inputs, input bit width, image size, and resource usage strategy can be configured. Pipelining and critical path optimization have been performed for the computing unit so that it can run at higher frequencies.

Our proposed methods can improve the computational speed of HE. Compared with the single-core CPU i7-8750H, the acceleration ratio of the PCIe3.0*16 interface can be up to 22.6 times higher. Compared with other FPGA-based implementations of single-input architectures (based on Zynq-XC7Z030) [16], the performance of the proposed architecture is 43.0 times higher.

Our work mainly aimed to achieve better algorithm acceleration performance rather than optimized resource utilization and power consumption. Therefore, in the performance comparison section, we only compared the acceleration ratio and frequency, without taking into account resource utilization and power consumption.

The current acceleration ratio is mainly limited by the speed of the IO interface. Secondary factors affecting the performance are the maximum BRAM operating frequency and the BRAM capacity. A single HE core can run at the maximum frequency of the FPGA (4k, 32 inputs, based on xcvu13p@891MHz), except for when BRAM effects are present.

Future improvements can use the latest PCIe interface. If we switch to a later version of PCIe, the acceleration ratio will be greater, ideally up to 90× based on PCIe5.0. Faster BRAM with a larger capacity can also be used to ensure that the whole circuit runs at a very high frequency.

## Figures and Tables

**Table 1:** Acceleration rate of the proposed method (PCIe*16) and other FPGA-based implementations (image size: 256 × 256).

Other FPGA-Based Implementations	Model of FPGA	Frequency (MHz)	Acceleration Rate (Utilizing CL, CL, and DSP)	Acceleration Rate (Utilizing CL, DSP, and BRAM)
Hazra et al. [16]	XC7V2000T	368	21.4×	43.0×
Alsuwailem et al. [17]	EP2S15F484C3	250	31.5×	63.3×
Sachdeva et al. [18]	unknown	250	31.5×	63.3×
Li et al. [20]	xc4vsx35-10ff668	200	39.4×	79.1×
Sadad et al. [21]	XC7A100T	100	78.7×	158.2×
Li et al. [22]	XC4010	50	1573.8×	3163.0×
Lianfa et al. [23]	Flex10k10	3.33	2360.7×	4744.5×
Salcic et al. [25]	Flex8000	30.0	262.3×	527.2×

## Data Availability

The data presented in this study are available on request from the corresponding author.
